# Glycopeptide Heteroresistance Among Coagulase-Negative Staphylococci: A Systematic Review and Meta-analysis

**DOI:** 10.1093/ofid/ofaf494

**Published:** 2025-08-14

**Authors:** Clark D Russell, Min Ke, Simon Dewar, Naomi J Gadsby, Ewan S Olson, Mandy Wootton

**Affiliations:** Centre for Inflammation Research, Institute for Regeneration and Repair, The University of Edinburgh, Edinburgh, UK; NHS Lothian Infection Service, Royal Infirmary of Edinburgh, Edinburgh, UK; Clinical Infection Research Group, Western General Hospital, Edinburgh, UK; NHS Lothian Infection Service, Royal Infirmary of Edinburgh, Edinburgh, UK; NHS Lothian Infection Service, Royal Infirmary of Edinburgh, Edinburgh, UK; Clinical Infection Research Group, Western General Hospital, Edinburgh, UK; NHS Lothian Infection Service, Royal Infirmary of Edinburgh, Edinburgh, UK; NHS Lothian Infection Service, Royal Infirmary of Edinburgh, Edinburgh, UK; Specialist Antimicrobial Chemotherapy Unit, Public Health Wales, University Hospital of Wales, Cardiff, UK

**Keywords:** coagulase negative staphylococci, glycopeptide heteroresistance, teicoplanin, vancomycin, endocarditis

## Abstract

We identified a high prevalence of glycopeptide heteroresistance amongst coagulase-negative staphylococci (41.4%, 95% confidence interval 30.7–52.9; meta-analysis including n = 1432 isolates). Heteroresistance was associated with methicillin resistance, did not require glycopeptide exposure, and may be more prevalent among isolates from invasive infections. Heteroresistance may represent an under-appreciated reason for treatment failure of CoNS infections.

Infections caused by coagulase-negative staphylococci (CoNS) can be difficult to treat due to the high capacity of these organisms to form biofilm and the frequent occurrence of multidrug resistance [1, 2]. In particular, CoNS are a common cause of infective endocarditis affecting both native and prosthetic valves, occurring in older patients with multimorbidity [3–5]. One-year mortality for CoNS and *Staphylococcus aureus* endocarditis is equivalent, underscoring the clinical importance of these organisms in invasive infections. The very high prevalence of methicillin resistance among CoNS has led to a reliance on glycopeptides for treatment. “Low-level” or “intermediate” (non-*vanA*) glycopeptide resistance in CoNS and *S. aureus* (glycopeptide intermediate *S. aureus*, “GISA”) can be identified based on minimum inhibitory concentration (MIC) thresholds, determined by broth microdilution (MIC 4–8 mg/L for GISA). The underlying mechanism is mutational and polygenic, associated with cell wall alterations [1, 6]. Glycopeptide heteroresistance is thought to be a precursor to homogenous “intermediate” resistance and describes an isolate which is considered susceptible based on standard MIC determination, but which contains a minority population (eg, 1 in 10^6^ cells) with an MIC out with the susceptible range, determined by population analysis profiling (PAP) [7, 8]. This represents a diagnostic challenge, since routinely used methods for MIC determination cannot definitively identify this resistance phenotype. Glycopeptide heteroresistance is estimated to be present in 1.3% of methicillin-resistant *S. aureus* (MRSA) isolates (referred to as “hGISA”), associated with deep-seated infections and glycopeptide treatment failure [9]. Considering the reliance on glycopeptides to treat invasive CoNS infections such as endocarditis, this occult resistance phenotype could be clinically important during the treatment of CoNS infections. We aimed to estimate the prevalence of glycopeptide heteroresistance in CoNS using data from published studies.

## METHODS

### Identification of Studies

We searched PubMed on 13 July 2024 and Embase on 2 May 2025 for articles published in English containing the terms (Staphylococcus (MeSH) OR “staphylococc*”) AND (“reduced susceptibility” OR “decreased susceptibility” OR “intermediate susceptibility” OR “heteroresistance” OR “heteroresistant” OR “vancomycin-intermediate” OR “heterogeneously resistant”) AND (“vancomycin” OR “teicoplanin” OR “glycopeptide*” OR Glycopeptides (MeSH)). Two authors (C. D. R. and M. K.) independently reviewed all search results and bibliographies to identify relevant studies (Supplementary Figure 1). We included studies reporting either the prevalence or clinical outcomes (compared with a control group) of glycopeptide heteroresistance in CoNS, determined by any testing method. We excluded studies restricted to a single antimicrobial resistance phenotype (eg, exclusively methicillin-resistant CoNS), restricted to isolates with raised glycopeptide MIC by standard determination, case reports, and studies of nonsystematically gathered isolates.

### Analysis

The ROBIS tool [10] was used for risk of bias assessment. Meta-analysis of the proportion of glycopeptide heteroresistance was performed with the *meta* package (version 7.0-0) in RStudio. A random effects model was chosen due to interstudy variability in patient populations, clinical syndromes, and CoNS species. Categorical variables were compared using Fisher's exact test (GraphPad Prism Version 10.3.1).

## RESULTS

### Identified Studies

**Figure 1.**
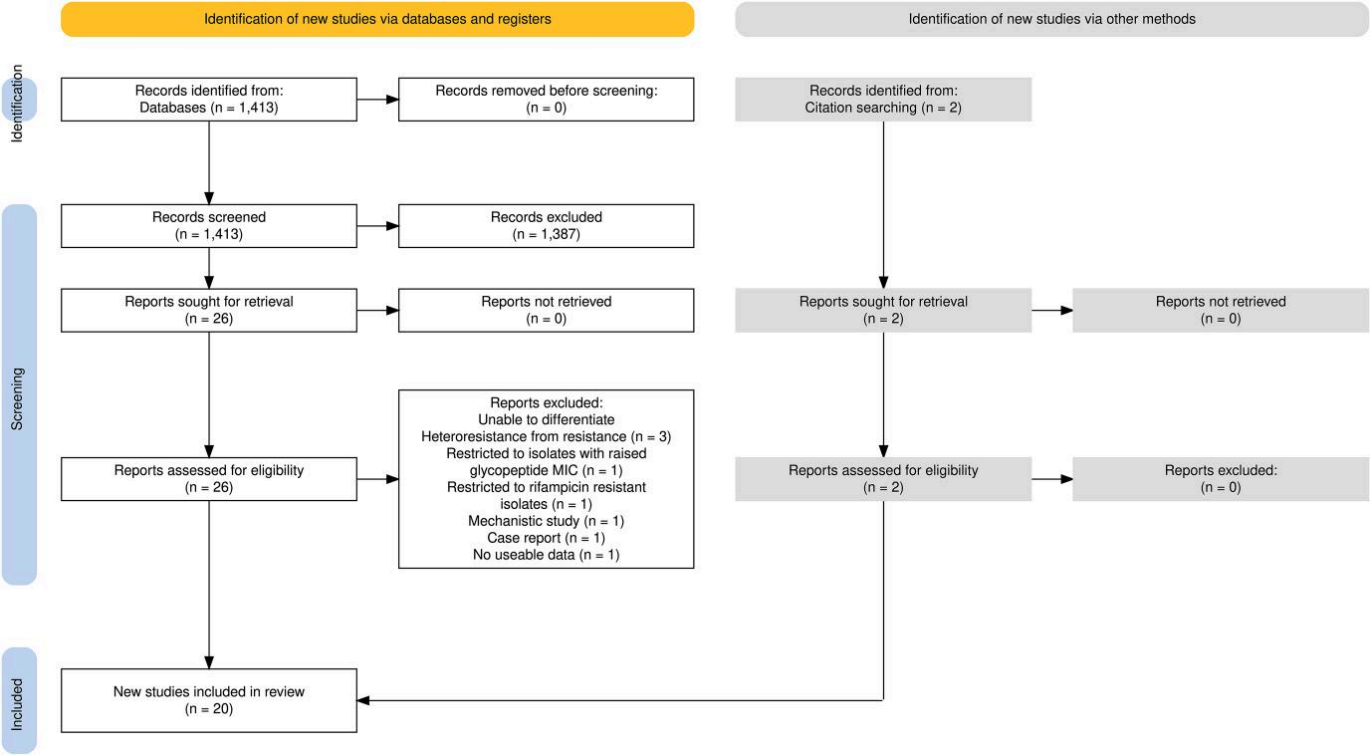
PRISMA diagram. Created using PRISMA2020 [11].

**Figure 2.**
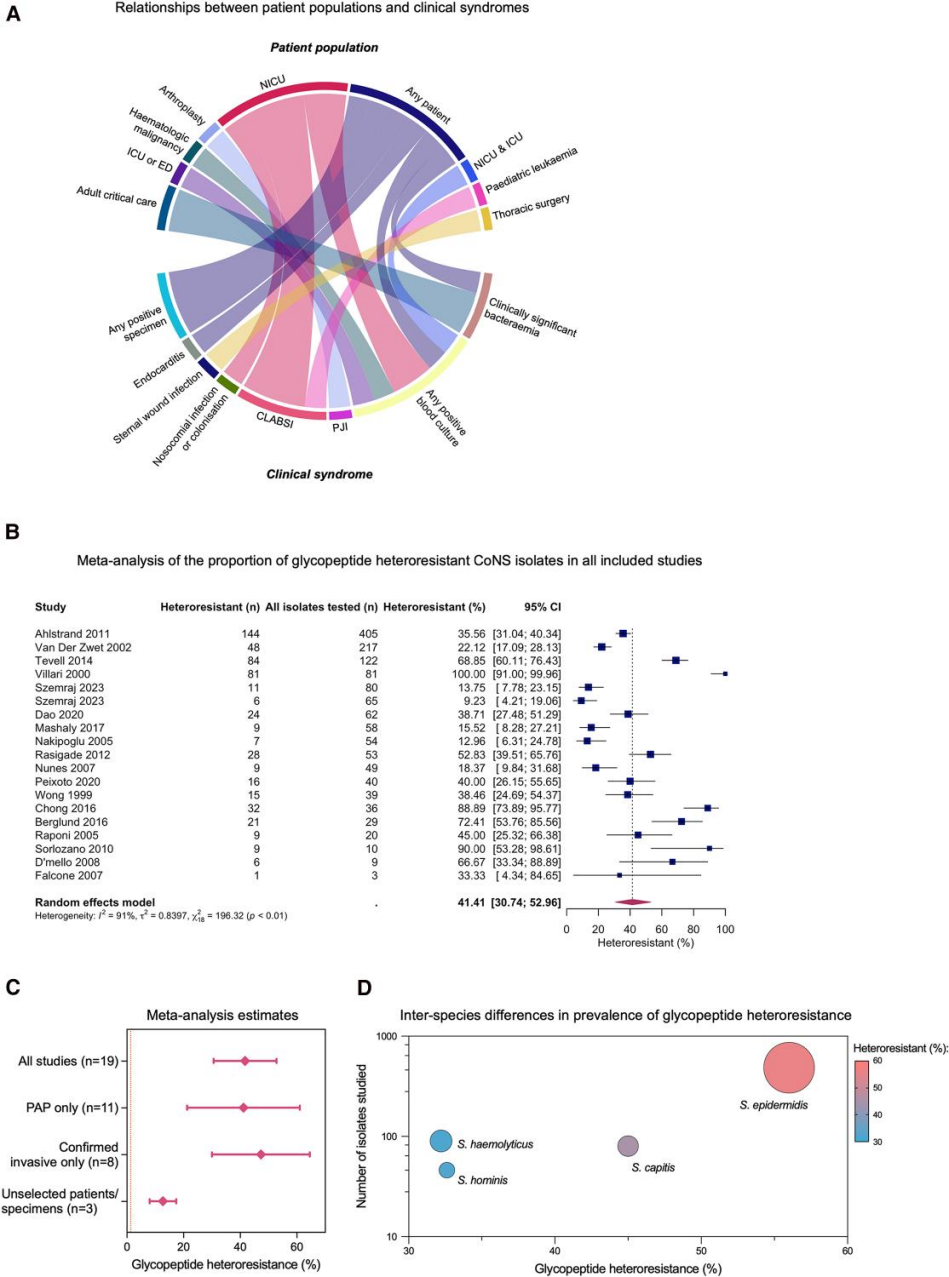
Meta-analysis of glycopeptide heteroresistance in coagulase-negative staphylococci. *A*, Circos plot visualiing the contribution of different patient populations to the clinical syndromes from which CoNS were recovered in the included studies. Connecting links are proportional to the number of studies. *B*, Forest plot of meta-analysis results. Boxes represent the prevalence results from individual studies, sized proportional to study size, and horizontal lines indicate 95% CIs for the individual studies. The diamond represents the combined prevalence estimate considering all studies, with the horizontal tips representing the 95% CI of the estimate. *C*, Comparison of glycopeptide heteroresistance prevalence estimates when meta-analysis includes all studies, only studies using PAP, only studies including isolates from confirmed invasive infections (clinically significant bacteremia, CLABSI, PJI, or endocarditis), or only studies including isolates from unselected patients and specimens. Diamonds represent prevalence estimates and bars represent 95% CI. N refers to number of included studies. Horizontal dotted line represents estimated prevalence of glycopeptide heteroresistance in MRSA from [9]. *D*, Bubble plot of interspecies differences in prevalence of glycopeptide heteroresistance. Shading of the bubble represents the percentage of heteroresistant isolates (*x*-axis). The size of the bubble represents the number of isolates studied for each species (*y*-axis, log_10_ scale). Abbreviations: NICU, neonatal intensive care unit; ICU, intensive care unit (adult); ED, emergency department; CLABSI, central line associated bloodstream infection; PAP, population analysis profiling; PJI, prosthetic joint infection; CI, confidence interval.

We identified 20 studies investigating glycopeptide heteroresistance in CoNS, using either vancomycin alone or both vancomycin and teicoplanin (Figure 1, Table 1). Nineteen studies reported data on prevalence and 2 reported information regarding clinical outcomes. Included studies evaluated a range of patient populations and clinical syndromes (Figure 2*A*). The results of our risk of bias assessment are shown in Supplementary Table 1. Bias was introduced because most studies were restricted to selected patient populations or syndromes (Figure 2*A*); only 3 studies reported results from any patient with any positive specimen. The CoNS isolates investigated were predominantly recovered from blood cultures though not all represented clinically significant bacteremia (Figure 2*A*). PAP was used for detection of heteroresistance (either as the only method, or as a confirmatory test) in 11/20 studies. Of the 9 studies not using PAP, 5 used the macro gradient strip test method and 4 used screening agar (brain heart infusion agar with 4 mg/L vancomycin) [32].

**Table 1.** Included Studies

First Author	Year	Country	Design	Patient Population	Syndrome(s)	Specimen Type(s)	Glycopeptide	Method 1	Method 2	Positive Control Strain	Data Available	N Isolates
Wong et al [12]	1999	China	Prospective	Any patient	Clinically significant bacteremia	Blood culture	Van	Screening agar^a^	MIC of colonies growing on screening agar	Not stated	Prevalence	39
Villari et al [13]	2000	Italy	Prospective	NICU	Nosocomial infection and colonization	Any	Van + Teic	PAP	None	Not stated	Prevalence	81
Van Der Zwet et al [14]	2002	The Netherlands	Retrospective	NICU	Any positive blood culture	Blood culture	Van	Screening agar^a^	None	Not stated	Prevalence	217
Nakipoglu et al [15]	2005	Turkey	Prospective	Any patient	Any positive specimen	Not stated	Van	Screening agar^a^	PAP	*S. aureus* Mu3	Prevalence	54
Raponi et al [16]	2005	Italy	Prospective	Adult critical care	Clinically significant bacteremia	Blood culture or catheter tip	Van + Teic	Macro *E*-test	None	Not stated	Prevalence	20
Falcone et al [17]	2007	Italy	Prospective	Any patient	Endocarditis	Blood culture	Van	PAP	None	Yes (not stated which)	Prevalence	3
Nunes et al [18]	2007	Brazil	Prospective	Any patient	Any positive blood culture	Blood culture	Van + Teic	Screening agar^a^	PAP	*S. aureus* Mu50	Prevalence	49
D'mello et al [19]	2008	Australia	Retrospective	NICU	Any positive blood culture	Blood culture	Van	Screening agar^a^	PAP	*S. aureus* Mu3	Prevalence	9
Sorlozano et al [20]	2010	Spain	Prospective	ICU or ED	Any positive blood culture	Blood culture	Van + Teic	Macro *E*-test	None	Not stated	Prevalence	10
Ahlstrand et al [21]	2011	Sweden	Prospective	Hematologic malignancy	Any positive blood culture	Blood culture	Van + Teic	Macro *E*-test	None	Not stated	Prevalence	405
Rasigade et al [22]	2012	France	Retrospective	NICU and ICU	Any positive blood culture	Blood culture	Van	Screening agar^a^	None	*S. aureus* Mu3 & Mu50	Prevalence	53
Tevell et al [23]	2014	Sweden	Prospective	Arthroplasty	Prosthetic joint infection	Tissue from arthroplasty	Van + Teic	Macro *E*-test	None	Not stated	Prevalence	122
Berglund et al [24]	2016	Sweden	Retrospective	Thoracic surgery	Sternal wound infection	Surgical tissue	Van	PAP	None	Not stated	Prevalence	29
Chong et al [25]	2016	Canada	Prospective	NICU	CLABSI	Blood culture	Van	Macro *E*-test	PAP	*S. aureus* Mu3	Prevalence	36
Blanchard et al [26]	2017	Canada	Retrospective	NICU	CLABSI	Blood culture	Van + Teic	Macro *E*-test	None	Not stated	Clinical	NA
Mashaly et al [27]	2017	Egypt	Prospective	Adult critical care	Clinically significant bacteremia	Blood culture	Van	Screening agar^a^	PAP	Not stated	Prevalence	58
Peixoto et al [28]	2020	Brazil	Prospective	NICU	CLABSI	Blood culture	Van	Screening agar^a^	None	Not stated	Prevalence	40
Dao et al [29]	2020	USA	Retrospective	Pediatric leukemia	CLABSI	Blood culture	Van	PAP	None	*S. aureus* Mu3	Prevalence Clinical	62
Szemraj et al [30]	2023	Poland	Retrospective	Any patient	Any positive specimen	Any	Van	Screening agar^a^	PAP	*S. aureus* Mu3	Prevalence	80
Szemraj et al [31]	2023	Poland	Retrospective	Any patient	Any positive specimen	Any	Van	Screening agar^a^	PAP	*S. aureus* Mu3	Prevalence	65

Abbreviations: NICU, neonatal intensive care unit; ICU, intensive care unit (adult); ED, emergency department; CLABSI, central line associated bloodstream infection; MIC, minimum inhibitory concentration; PAP, population analysis profile.; Van, vancomycin; Teic, teicoplanin.

^a^Brain heart infusion agar with 4 mg/L vancomycin.

### Prevalence of Glycopeptide Heteroresistance

Considering all studies reporting data on prevalence (including 1432 isolates; Figure 2*B*), 41.41% of isolates (95% confidence interval [CI] 30.74–52.96) exhibited heteroresistance, with evidence of substantial heterogeneity (*I*^2^ = 91%). A very similar estimate was obtained when the analysis was restricted to studies using the PAP method (40.06%, 95% CI 21.82–61.54, *I*^2^ = 91%, n = 526 isolates; Figure 2*C*; Supplementary Figure 1). Considering only studies investigating CoNS isolates recovered from confirmed invasive infections (clinically significant bacteremia, CLABSI, PJI, or endocarditis), 47.05% were estimated to exhibit heteroresistance (95% CI 30.14–64.67, *I*^2^ = 89%, n = 380 isolates; Figure 2*C*; Supplementary Figure 2). However, when restricted to the 3 studies including isolates from any specimen from any patient, a lower estimate was obtained (12.2%, 95% CI 8.3–17.6, n = 199 isolates; Supplementary Figure 3) without heterogeneity (*I*^2^  *=* 0%).

Methicillin susceptibility/resistance was reported for 357/1432 isolates. Detection of glycopeptide heteroresistance was positively associated with methicillin resistance (138/285 methicillin-resistant vs 23/72 methicillin-susceptible isolates, odds ratio = 2.0, 95% CI 1.2–3.5). Species level identification was reported for 700/1432 isolates, predominantly *Staphylococcus epidermidis* (n = 484). Interspecies differences in prevalence were apparent when glycopeptide heteroresistance was stratified by species (Figure 2*D*). This phenotype was identified in 56.0% (271/484) of *S. epidermidis* isolates, 45.0% (36/80) of *Staphylococcus capitis* isolates, 32.6% (15/46) of *Staphylococcus hominis* isolates, and 32.3% (29/90) of *Staphylococcus haemolyticus* isolates (*P* < .0001).

### Prior Glycopeptide Exposure

Patient-level glycopeptide exposure was reported by 6 studies, including isolates from 65 patients. Forty-six of the 65 patients had no documented prior glycopeptide exposure. One additional study quantified days of prior vancomycin exposure in the 60 days preceding CoNS isolation and compared this between patients with a susceptible versus heteroresistant isolate [29]. In this study, isolation of a heteroresistant isolate was not associated with overall vancomycin exposure but was positively associated with prophylactic vancomycin exposure.

### Clinical Implications of Glycopeptide Heteroresistance

Two individual studies reported findings related to the clinical implications of glycopeptide heteroresistance, which we summarize here. Among a subset of 40 episodes of pediatric CLABSI caused by CoNS in a retrospective observational study, glycopeptide heteroresistance was associated with treatment failure (death or relapse) and poor clinical response (treatment failure or persistent bacteremia after vancomycin initiation) [29]. These associations were independent of patient age or isolate vancomycin MIC, and were replicated in a subgroup analysis restricted to *S. epidermidis*. Therapeutic implications of heteroresistance were investigated in a retrospective observational study of CLABSI caused by glycopeptide heteroresistant CoNS (predominantly *S. epidermidis*) in a neonatal intensive care unit (NICU) [26]. Eighty-nine patients were included, 33 treated with linezolid and 56 with vancomycin. Numerically, linezolid was associated with a reduction in late recurrences (0/33 vs 8/56, univariate *P* = .02). No statistical difference was seen in a multivariate analysis but the small sample size and low event rate mean a true difference cannot be excluded.

## DISCUSSION

We have identified a high prevalence of glycopeptide heteroresistance among CoNS. We obtained a lower prevalence estimate when restricting to studies of unselected patients/specimens, indicating potential selection bias and/or enrichment of this phenotype in isolates from invasive CoNS infections. However, using either estimate, this resistance phenotype appears substantially more prevalent among CoNS compared with MRSA [9]. The available data regarding the clinical implications of glycopeptide heteroresistance are limited, but the findings of 2 observational studies suggest this phenotype could be clinically important, like it is for MRSA infection [9].

In studies where prior vancomycin exposure was investigated, this was documented in less than one-third of cases, suggesting it is not a requirement for heteroresistance. Bloodstream isolates of CoNS and *S. aureus* recovered from patients with endocarditis exhibit resistance to killing by platelet microbicidal protein (PMP) in vitro, and this finding is associated with co-occurrence of the hGISA phenotype in *S. aureus* [33, 34]. In a rabbit model of *S. aureus* PJI, PMP and daptomycin cross-resistance emerged even in the absence of daptomycin exposure [35]. Overall, it is possible that glycopeptide heteroresistance represents an adaptation to host innate immunity, rather than being an exclusively microbiologic phenomenon. This would also be consistent with the higher prevalence of this phenotype among isolates recovered from invasive infection compared with unselected isolates.

Several limitations to our analysis underscore the need for further investigation. First, there is no universally recognized method of identification of glycopeptide heteroresistance in CoNS. Guidance for the detection of hGISA exists, for example, from the European Committee on Antimicrobial Susceptibility Testing [36]. The recommended approach involves a screening test such as the macrogradient strip test, followed by confirmatory PAP, using the Mu3 strain as a positive control. PAP is technically difficult and usually performed in reference laboratories. A range of approaches were used to identify glycopeptide heteroresistance in the studies included in our meta-analysis, but a very similar prevalence estimate was obtained when restricting the analysis to studies using PAP. These methods have not been extensively investigated for CoNS, and there is currently no recognized positive control CoNS strain, though existing data support their usage for CoNS [32]. The vancomycin breakpoint for CoNS is 1 doubling dilution higher than for *S. aureus* (4 vs 2 mg/L), leading to potential overcalling of heteroresistance if porting the same method used for detection of hGISA. Second, there was a very high degree of heterogeneity determined by the *I*^2^ statistic, potentially related to differences between selected versus unselected patient cohorts included. Third, a source of bias in included studies is the contribution of cross-transmission of strains between patients within the same center impacting on prevalence estimates. For example, 100% of isolates included in a study conducted in a NICU demonstrated heteroresistance, probably due to cross-transmission [13].

In conclusion, glycopeptide heteroresistance in CoNS may be an under-appreciated additional challenge when using glycopeptides to treat deep-seated infections caused by these organisms. Prospective case–control studies, using comparable antimicrobial susceptibility testing methods, and including appropriate control groups, are required to definitively determine the impact of this occult resistance phenotype on clinical outcomes. Considering the existence of glycopeptide/daptomycin cross-resistance in *S. aureus*, and emergence of daptomycin heteroresistance during vancomycin therapy, the impact on daptomycin treatment outcomes should also be evaluated [37, 38]. In the meantime, while acknowledging the technical and logistical challenges of doing so, we suggest that testing for heteroresistance should be considered if there is concern for treatment failure during treatment of CoNS infections with glycopeptides, especially if the focus of infection is nonremovable. Greater familiarity with the macrogradient strip test screening method would facilitate the identification of potentially heteroresistant isolates for referral to a specialist laboratory for PAP. This will require availability of appropriate and standardized positive control strains of CoNS, and an external quality assurance scheme.

## Supplementary Material

ofaf494_Supplementary_Data

## Supplementary Data

Supplementary materials are available at *Open Forum Infectious Diseases* online. Consisting of data provided by the authors to benefit the reader, the posted materials are not copyedited and are the sole responsibility of the authors, so questions or comments should be addressed to the corresponding author.
