# Supplementary material for: Glycopeptide Heteroresistance Among Coagulase-Negative Staphylococci: A Systematic Review and Meta-analysis
Source: Open Forum Infect Dis. 2025 Aug 14;12(8):ofaf494. doi: 10.1093/ofid/ofaf494 (PMC12378235; doi:10.1093/ofid/ofaf494)
Supplement: ofaf494_Supplementary_Data [file ofaf494_supplementary_data.pdf]

Supplementary Material

Contents:

Table S1: Risk of bias assessed using ROBIS method..2

Figure S1: Forest plot of sub-group of studies using PAP method to identify heteroresistance 3

Figure S2: Forest plot of sub-group of studies investigating isolates recovered from confirmed invasive infections..4

Figure S3: Forest plot of sub-group of studies investigating isolates recovered from unselected patients and specimens..5

Table S1: Risk of bias assessed using ROBIS method

Eligibility criteria	Identification and selection	Data collection and study appraisal	Synthesis and findings
Low	Low	Low	High

Study	Heteroresistant (n)	All isolates tested (n)	Heteroresistant (%)	95% CI
Nakipoglu 2005	7	54	12.96	[6.31; 24.78]
Nunes 2007	9	49	18.37	[9.84; 31.68]
Berglund 2016	21	29	72.41	[53.76; 85.56]
Mashaly 2017	9	58	15.52	[8.28; 27.21]
Dao 2020	24	62	38.71	[27.48; 51.29]
Szemraj 2023	11	80	13.75	[7.78; 23.15]
Villari 2000	81	81	100.00	[91.00; 99.96]
Falcone 2007	1	3	33.33	[4.34; 84.65]
D'mello 2008	6	9	66.67	[33.34; 88.89]
Chong 2016	32	36	88.89	[73.89; 95.77]
Szemraj 2023	6	65	9.23	[4.21; 19.06]
**Random effects model**		.	**40.06**	**[21.82; 61.54]**

Heterogeneity: $I^2 = 91\%$, $\tau^2 = 1.8237$, $\chi^2_{10} = 109.79$ ($p < 0.01$)

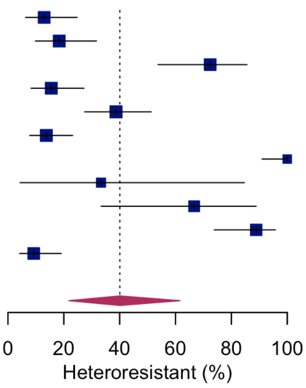

Figure S1: Forest plot of sub-group of studies using PAP method to identify heteroresistance

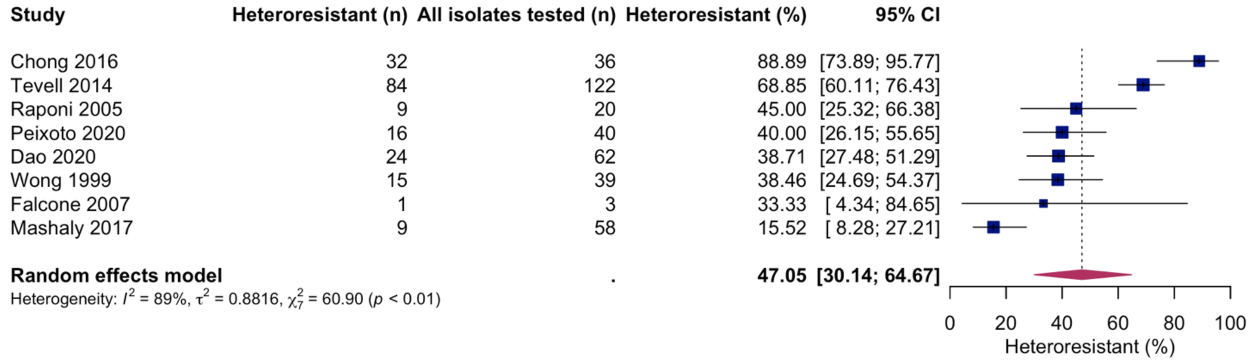

Figure S2: Forest plot of sub-group of studies investigating isolates recovered from confirmed invasive infections

Confirmed invasive infections were clinically significant bacteraemia, central line associated bloodstream infection, prosthetic joint infection, and endocarditis.

Study	Heteroresistant (n)	All isolates tested (n)	Heteroresistant (%)	95% CI
Szemraj 2023	11	80	13.75	[7.78; 23.15]
Szemraj 2023	6	65	9.23	[4.21; 19.06]
Nakipoglu 2005	7	54	12.96	[6.31; 24.78]
**Random effects model**		.	**12.21**	**[8.32; 17.58]**

Heterogeneity: $I^2 = 0\%$, $\tau^2 = 0$, $\chi^2_2 = 0.74$ ($p = 0.69$)

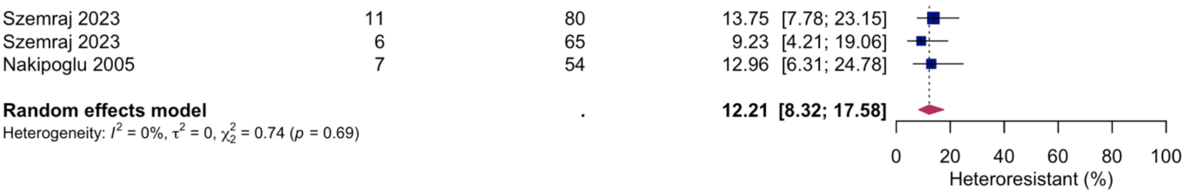

Figure S3: Forest plot of sub-group of studies investigating isolates recovered from unselected patients and specimens
